# Implementation of TasP: challenges and opportunities

**DOI:** 10.1186/1471-2334-14-S2-S8

**Published:** 2014-05-23

**Authors:** Joep Lange

**Affiliations:** 1 Department of Global Health, Academic Medical Center, University of Amsterdam, Amsterdam, 1012WX, The Netherlands

## 

Treatment as prevention (TasP) not only stands for prevention of HIV transmission by treating HIV-positives, but also for prevention of morbidity and mortality in those positives. Mathematical models have shown that it should be possible to “eradicate” HIV at the population level, or at least come close to it, if this approach were rigorously applied.

But these optimistic models are based on assumptions which are not realistic:

• That it is possible to test the whole adult population every year;

• That every individual found to be HIV positive will accept immediate antiretroviral treatment;

• That those accepting antiretroviral treatment will all be therapy adherent;

• That there will be no drug stock-outs;

• That there will be no development of antiretroviral drug resistance.

We should realize, however, that early treatment is good for an individual’s own benefit, and that this is the overriding reason to test as many people as possible and offer them antiretrovirals.

Early treatment prolongs life expectancy, prevents co-morbidities, and allows for task shifting from doctors to nurses and community workers. Moreover it will reduce TB incidence, which is not a trivial fact in resource poor settings. Early treatment thus presents an enormous opportunity to save lives, reduce HIV transmission and tackle the dual epidemic of HIV and TB. The world must be willing, however, to surmount the enormous hurdles (financial, logistical) that stand in the way of realization of this opportunity.

